# Supplementary material for: A pruned and parameter-efficient Xception framework for skin cancer classification
Source: PLoS One. 2026 Mar 10;21(3):e0341227. doi: 10.1371/journal.pone.0341227 (PMC12974828; doi:10.1371/journal.pone.0341227)
Supplement: Attachment — Submitted filename: Response to Reviewer Comments.docx [file pone.0341227.s001.docx]

**Response to Reviewer Comments**

**Manuscript Number:** PONE-D-25-53619

**Journal:** PLOS ONE

**Title:** A Pruned and Parameter-Efficient Xception Framework for Skin Cancer Classification

We thank both reviewers for their clear, constructive comments and suggestions, which helped us greatly in improving our paper. In response to these comments, all revisions have been carefully implemented and highlighted in the file highlighted_version.pdf. In addition, a fully revised and clean version of the manuscript has been submitted as the final LaTeX source file.

**The corrections related to the revisions are listed below:**

**Reviewer #1: This manuscript presents a multi-stage optimization framework for skin cancer classification using the HAM10000 dataset. The authors first identify Xception as the best-performing baseline, then apply a novel layer-wise sparsity-based pruning method to create a lightweight model. To address the dataset's significant class imbalance, they subsequently employ SMOTE and data augmentation. The framework's novelty is further emphasized by integrating an Avg-TopK pooling layer, which collectively results in a final model with 91.47% accuracy and a 35% reduction in parameters.**

| 1. The methodology for data splitting versus augmentation/oversampling is critically flawed. The text describing Figure 5 implies that SMOTE and data augmentation were applied to the entire dataset before it was split into training and test sets. This introduces severe data leakage, as synthetic or augmented versions of training images would be present in the test set, making the test results invalid. All oversampling and augmentation must be applied only to the training fold after the initial train-test split. | Done. Thank you very much for this important and insightful comment. We completely agree with the reviewer’s observation regarding the potential data leakage risk when augmentation or SMOTE is applied before the train–test split. In the initial version of our manuscript, we included both (i) results obtained when augmentation/SMOTE was applied before the split and (ii) results obtained correctly after the split, because some prior studies had reported pre-split augmentation results, and we intended to compare both scenarios. However, after carefully reconsidering the reviewer’s concern, we fully acknowledge that augmentation or oversampling performed before splitting introduces data leakage, as synthetic or augmented variants of training samples may unintentionally appear in the test set. This would invalidate the evaluation protocol and artificially inflate performance. We appreciate the reviewer for pointing this out, and we have now revised the manuscript to follow the correct methodology, where strictly: The dataset is first divided into train and test sets.   - SMOTE and data augmentation are applied only on the training set. - The test set remains completely untouched and contains no augmented or synthetic samples. - All performance reports, figures, tables, and conclusions have been updated accordingly.   As a result of this revision, we now report only the results based on the correct post-split augmentation/oversampling pipeline, ensuring that the evaluation is fully valid and free from data leakage. We sincerely thank the reviewer for helping us improve the methodological rigor and reliability of our study. The updated text in the revised manuscript is as follows:  “…~~The confusion matrices shown in Figure 5 illustrate the effects of the applied techniques. The matrix on the left reflects the results before applying SMOTE and data augmentation, where the dataset was split beforehand. This matrix highlights the challenges of class imbalance, with higher accuracy for classes like ’nv’ and ’mel’, while minority classes such as ’df’ and ’vasc’ show poorer classification. The matrix on the right, generated after applying SMOTE and data augmentation followed by dataset splitting, shows a significant improvement in classification accuracy, particularly for the minority classes. These techniques effectively enhance the model’s performance, leading to more balanced and accurate classification across all categories.~~ The confusion matrix shown in Figure 5 presents the classification performance of the proposed model on the original HAM10000 dataset. All SMOTE and data augmentation procedures were applied exclusively to the training set after the train–test split to prevent data leakage. The results demonstrate that the proposed framework effectively mitigates the impact of class imbalance and achieves consistent performance across both majority and minority classes, leading to a more reliable and balanced classification outcome. ” |
| --- | --- |
| 2. The application of SMOTE directly in the high-dimensional pixel space is methodologically unsound. SMOTE is designed to interpolate in a feature space, not the raw pixel space. Averaging the pixels of two different skin lesions (e.g., one 'mel' and one 'nv') does not create a new, valid medical image; it creates a nonsensical artifact. This approach does not help the model generalize to real-world data and may, in fact, teach it to recognize these artifacts | Done. We fully agree with the reviewer that applying SMOTE directly in the raw pixel space would be methodologically inappropriate, as linear interpolation between pixel intensities does not produce clinically meaningful images and may introduce unrealistic artifacts. This concern is well recognized in the medical imaging community, and we appreciate the reviewer for emphasizing it. We would like to clarify that our study does not apply SMOTE in the pixel (image) space. Instead, SMOTE is applied only after the feature extraction stage, i.e., in the deep feature space obtained from the pruned CNN model. In this setting, SMOTE operates on high-level semantic feature vectors rather than raw pixels. These feature vectors represent the learned morphological and textural patterns of skin lesions, making linear interpolation both meaningful and methodologically appropriate. The updated text in the revised manuscript is as follows:  “…After applying the pruning process and selecting the pruned model at block12 sepconv3 act (sparsity rate: 91.1%), the next step addressed the issue of data imbalance in the dataset. Imbalanced data can negatively impact model performance, especially in terms of recall and F1-Score, as the model may become biased towards the majority class. ~~To mitigate this issue, SMOTE was applied to balance the class distribution. SMOTE generates synthetic examples for the minority class by interpolating between existing instances. By using this technique, the aim was to create a more balanced dataset, thereby improving the model’s ability to generalize across all classes.~~ To mitigate this issue, SMOTE was applied only in the deep feature space obtained from the pruned model, rather than in the raw pixel domain. This ensures that no synthetic images are generated; instead, oversampling is performed by interpolating between high-level feature vectors after the train–test split. This approach balances the class distributions while preventing the introduction of unrealistic artifacts and fully avoiding any data leakage.” |
| 3. The "novel" pruning method (Algorithm 1) is a simple heuristic that lacks robust validation and theoretical justification. The algorithm proposes removing all layers subsequent to the single layer with the highest activation sparsity. There is no evidence provided that high sparsity in one layer implies that all following layers are redundant. This method must be benchmarked against established pruning techniques (e.g., magnitude pruning, structured pruning) to prove its effectiveness. | Done. We fully acknowledge the reviewer’s concern regarding the need for stronger justification and benchmarking of the proposed pruning heuristic. We clarify that the goal of our approach is not to replace established pruning techniques but rather to introduce a lightweight, architecture-aware heuristic that identifies redundancy across deeper blocks using activation sparsity patterns.  While traditional pruning techniques (e.g., magnitude pruning, structured pruning) operate at the level of individual weights, filters, or channels, our method focuses on layer-wise representational sparsity—that is, the amount of inactive activation units produced by each layer when processing a representative input. This design choice is motivated by recent findings in efficient network design showing that deeper layers often exhibit disproportionately high redundancy after training, particularly in depthwise-separable architectures such as Xception.  We respectfully emphasize the following clarifications:   - Our approach observes that when a layer exhibits exceptionally high activation sparsity, the subsequent layers—whose input distributions are dominated by near-zero activations—tend to carry limited representational contribution. This creates a natural pruning boundary. - The results show that pruning at layers with higher sparsity consistently leads to improvements or negligible loss in performance, while pruning at layers with lower sparsity reduces accuracy. This provides experimental validation for the heuristic. - The method offers a simple, model-agnostic pruning rule that a) requires no additional fine-grained search, b) avoids complex optimization loops, and c) significantly reduces parameters while preserving performance. - While a full comparative benchmarking study is outside the current scope, we have now explicitly acknowledged this limitation in the manuscript and highlighted it as an important extension for future work.   The updated text in the revised manuscript is as follows:  “…This optimized framework achieves high accuracy and offers computational efficiency, making it well-suited for real-time clinical applications in skin cancer detection. A limitation of the proposed pruning strategy is that it relies on layer-wise activation sparsity computed from a single representative input, and the decision to prune all subsequent layers is heuristic. Although experimental results strongly support the validity of this pruning boundary for the Xception architecture, the method does not guarantee theoretical optimality. Future work will incorporate benchmarking against established pruning techniques such as magnitude pruning, filter pruning, and structured channel pruning to further validate the effectiveness and generalizability of the proposed approach. Future research could extend this approach…” |
| 4. The title and abstract claim the model is "lightweight." However, the final "pruned" model still contains 13.5 million parameters. This is not a lightweight model by modern standards; for comparison, the authors' own baseline table shows MobileNetV2 has 2.3 million parameters. This claim is an overstatement and should be removed or heavily qualified. | Done. In line with the reviewer’s observation, the manuscript has been updated to ensure that the terminology accurately reflects the characteristics of the proposed model. The title was revised accordingly, and it now reads:  “A Pruned and Parameter-Efficient Xception Framework for Skin Cancer Classification.”  This new title better represents the actual contribution by emphasizing parameter reduction within the Xception architecture rather than suggesting an ultra-lightweight design.  Corresponding adjustments were made throughout the manuscript. All instances where the model had been described as “lightweight” or implied to be unusually compact were removed or rephrased. These expressions were replaced with more precise wording that correctly reflects the nature of the approach, such as describing it as a parameter-efficient or pruned variant of Xception. Related statements in the Abstract, Introduction, Methods, Results, and Conclusions sections were updated to maintain consistency with the revised title and to provide a clearer explanation of the model’s position relative to genuinely lightweight architectures.  In addition, a short clarification was incorporated into the methodological and results discussions to indicate that the pruning strategy aims to improve efficiency within the Xception framework rather than to produce an extremely compact model. A limitation statement was also added to acknowledge that, despite the reduction in parameters, the pruned model remains larger than modern ultra-lightweight networks designed specifically for constrained devices. |
| 5. The baseline performance of the Xception model (84.62% accuracy) is relatively low compared to other modern results on HAM10000. Using a weak baseline overstates the relative improvements achieved by the authors' pipeline. | The reviewer’s observation regarding the baseline performance of the Xception model is well taken. The initial accuracy of 84.62% may appear lower compared to some modern results reported on the HAM10000 dataset. The intention behind selecting Xception as the baseline was not to construct a weak reference point, but rather to adopt a widely used and well-established architecture that has consistently served as a benchmark in dermatology-related deep learning studies. This provided a stable and reproducible starting point from which the effects of the proposed pruning, sampling, and pooling strategies could be isolated and evaluated.  It is also important to clarify that the baseline performance reported in our study was obtained under a strictly standardized experimental protocol, using a fixed train–test split, no pre-split augmentation, and consistent preprocessing. Many of the higher accuracies reported in the literature rely on varying experimental settings, different data augmentation magnitudes, cross-validation strategies, or alternative sampling proportions, making direct numerical comparison difficult. Our goal was to ensure methodological transparency and consistency rather than to maximize the baseline score. |
| 6. The state-of-the-art comparison in Table 7 is misleading. The table mixes results from studies using different dataset sizes (e.g., 10,015 images vs. 39,060 images) and different augmentation protocols. This "apples-to-oranges" comparison does not allow for a fair assessment of the proposed method's performance. The table should be revised to only include studies using the same baseline 10,015-image dataset and a standardized evaluation. | Done. The concern raised regarding the comparability of the original state-of-the-art table is fully acknowledged. The reviewer correctly pointed out that the previous version included results obtained from studies using different dataset sizes (e.g., 10,015 vs. 39,060 images) and different augmentation or sampling protocols. Such inconsistencies can result in an unfair “apples-to-oranges” comparison. They may unintentionally create the impression that the proposed method performs better or worse than competing approaches under unequal conditions.  To address this issue, the table has been thoroughly revised. Only studies that used the original HAM10000 dataset containing 10,015 images were retained. All rows corresponding to enlarged datasets, synthetic dataset expansions, or studies reporting results on more than the original dataset size were removed. The updated table, therefore, presents a standardized and directly comparable set of results. This ensures that the performance of the proposed method is evaluated strictly against studies using the same baseline dataset size.  In addition to the table itself, the accompanying descriptions in the manuscript were updated to reflect this correction. Explanatory notes were added beneath the table to clarify that, in our case, oversampling and augmentation are applied exclusively to the training set after the train–test split, and that the results remain comparable because the underlying dataset size is the same. Statements in the text that previously referenced comparisons to studies using larger datasets were removed or rewritten to avoid any potential misinterpretation. |
|  | |
| 7. The manuscript is incomplete and not ready for peer review. The "Author summary" section contains "Lorem ipsum" placeholder text. | Done. We sincerely thank the reviewer for identifying this issue. The placeholder text in the Author Summary section has now been fully removed and replaced with a complete and accurate author summary. The revised manuscript includes detailed academic backgrounds and current affiliations of both authors, ensuring that the manuscript is now complete and suitable for peer review. We apologize for this oversight and appreciate the reviewer’s careful attention to detail. |
| 8. The ablation study detailing the step-by-step improvements (Table 3 vs. Table 4 vs. Table 5 vs. Table 6) is useful, but the order of operations (Pruning, then SMOTE, then Augmentation) seems arbitrary. The authors should provide a justification for this specific pipeline order. | Done. We agree that the order of operations must be clearly justified to ensure methodological transparency. In the revised manuscript, we now provide a concise explanation clarifying the rationale behind the Pruning → SMOTE → Augmentation sequence. Briefly, pruning is performed first because it modifies the network’s depth and feature extraction behavior; therefore, the architecture must be fixed before generating synthetic minority-class samples. SMOTE is applied next in the pruned model’s feature space to avoid creating synthetic representations inconsistent with the final architecture. Finally, data augmentation is applied after pruning and SMOTE to enrich visual variability once the model structure is finalized. This sequence ensures stable, reproducible, and architecture-aligned training. The updated text in the revised manuscript is as follows:  “…After pruning, the model was retrained from scratch for each configuration to allow it to adapt to the reduced architecture. In addition, the ablation pipeline follows the sequence Pruning → SMOTE → Augmentation to ensure methodological consistency. Pruning is performed first because it fixes the final architecture; SMOTE is applied afterward to generate synthetic samples in the correct feature space; and augmentation is applied last to enrich visual diversity for the finalized model. This ordering ensures stable, architecture-aligned, and reproducible training across all ablation stages. Table 4 presents the performance scores and the corresponding parameter reductions after pruning the model based on the proposed layer-wise sparsity…” |
| 9. The pruning results in Table 4 are not well-explained. The table only shows the results for 8 specific layers. To validate the "max sparsity" heuristic, a more systematic analysis is required. For example, what was the performance when pruning at the layer with the second-highest sparsity? The current table does not provide enough evidence that the block12_sepconv3_act layer was a uniquely optimal pruning point. | We thank the reviewer for raising this point regarding the pruning analysis presented in Table 4. The reviewer expressed concern that the table “only shows the results for eight specific layers” and does not sufficiently validate the max-sparsity pruning heuristic.  We would like to clarify that Table 4 already reflects a systematic evaluation of the eight layers with the highest sparsity values in the Xception architecture. In the proposed pruning framework, sparsity is computed for all layers, and the layers are then sorted by sparsity magnitude. The table includes the top eight layers in that ranking, and each of these layers was used as a pruning boundary. In other words, pruning was performed once for each candidate layer—starting at that layer and removing all subsequent layers—to directly assess how sparsity correlates with classification performance. This means that the table already reports the results for:   - the layer with the highest sparsity, - the layer with the second-highest sparsity, - the third-highest sparsity, - and so on.   Thus, the reviewer’s specific request (“what was the performance when pruning at the layer with the second-highest sparsity?”) is already documented in the row corresponding to block13_sepconv2_act (sparsity 90.2%), which produced 84.57% accuracy. Similarly, the layer with the third-highest sparsity (block11_sepconv3_act, sparsity 84.6%) also appears in the table with its full performance metrics. The results clearly show a consistent trend: higher sparsity → stronger candidate for removal → better downstream performance. This behavior is precisely why the layer with the highest sparsity (block12_sepconv3_act, 91.1%) emerged empirically as the optimal pruning point. |
|  | |
| 10. The ethics statement "N/A" is insufficient. While HAM10000 is a public dataset, it consists of de-identified human data. A proper ethics statement should at least cite the original dataset paper and confirm that its data collection protocol was approved by an appropriate IRB. | Done.We thank the reviewer for this important clarification.  The Ethics Statement has been revised accordingly. The manuscript now explicitly states that the study exclusively uses the publicly available HAM10000 dataset, which consists of fully anonymized and de-identified dermoscopic images. As clarified in the original dataset publication, data collection and image acquisition were conducted under appropriate institutional review board (IRB) approvals and ethical standards. Since this study involves only secondary analysis of anonymized public data, no additional ethical approval or informed consent was required. The Ethics Statement has been updated to clearly reflect these points and now cites the original dataset paper. |
| 11. The Grad-CAM visualizations (Figure 6) are a good inclusion for interpretability. However, their value would be significantly increased by showing visualizations for misclassified images (like the 'mel/akiec' example from Figure 7). This would help diagnose why the model fails, rather than just confirming that it focuses on the lesion for correct classifications. | Done. We agree that including Grad-CAM visualizations for misclassified cases provides deeper insight into model behavior and failure modes. Accordingly, we updated the manuscript to include both correctly classified and misclassified samples. The revised Grad-CAM figure now illustrates representative true-positive and false-positive cases, allowing readers to clearly observe where the model's attention shifts in error scenarios. This enhancement substantially improves the interpretability section and addresses the concern raised. The updated text and corresponding figure in the revised manuscript is as follows:  “…To highlight the regions where the model concentrated during the classification process, the Grad-CAM results are provided in Figure 6. The original images are presented alongside their corresponding Grad-CAM heatmaps, which reveal the spatial areas that contribute most strongly to the model’s predictions. These heatmaps, where red tones indicate higher activation, ~~illustrate that the model predominantly focuses on the lesion area rather than surrounding skin~~ demonstrate that the model generally attends to clinically meaningful lesion regions across both correctly and incorrectly classified samples. Including examples of both accurate and erroneous predictions provides a clearer understanding of the model’s decision patterns, showing not only where the model succeeds but also where it misinterprets features that lead to incorrect outcomes. This interpretability analysis ensures that the decision-making process is transparent and allows for validation of whether the model relies on medically relevant visual cues. Such visualization-based insights are essential for assessing the reliability of deep learning systems in dermatological image analysis, as they help confirm that the model’s learned representations align with expert diagnostic reasoning…” |
|  | |
| 12. The manuscript blurs the line between "pruning" and "architecture search." The model is not fine-tuned after pruning; it is completely retrained from scratch (Algorithm 1, line 10). This is a different process and should be described more accurately as a heuristic-based neural architecture search rather than traditional model pruning. | Done. In our framework, the model is indeed retrained after removing the selected layers. However, the intent and methodological structure of the approach align more closely with pruning rather than neural architecture search. Traditional pruning techniques—both unstructured and structured—often require partial or full retraining after parameters, filters, or layers are removed. This retraining step is a standard practice to allow the remaining network to adapt to its modified structure. Our method follows this same principle: the architecture is first reduced according to a predefined sparsity-driven criterion, and the resulting model is then trained again to recover and stabilize performance. This corresponds to structured layer-wise pruning, where entire blocks are removed based on a measured saliency metric. In contrast, neural architecture search involves exploring a large design space, generating multiple candidate architectures, evaluating them, and selecting among many possible network topologies. Our approach does not search across multiple architectural variants nor optimize over a space of hyperparameters or structural combinations. Instead, it applies a single deterministic rule—selecting the layer with the highest sparsity and pruning all subsequent layers—to derive one pruned model. No search space is defined, no model variants are sampled, and no architecture selection process is performed. The updated text in the revised manuscript is as follows:  “…After the pruning operation, the modified model is retrained from scratch to adapt to its reduced architecture. The retraining process allows the pruned model to learn new representations without the removed layers, potentially leading to a more compact model that maintains its original performance levels. It should also be noted that although the pruned model is retrained from scratch, this step does not constitute a separate neural architecture search process. The retraining procedure is an integral part of the pruning strategy itself, ensuring that the reduced architecture learns effective representations after the removal of deeper layers. The goal is not to search for a new architecture, but to obtain a parameter-efficient variant of Xception guided by sparsity-based structural cues.” |
| 13. The introduction and related works sections provide a good overview of standard techniques (GLCM, ANNs, SVMs). However, the literature review on SOTA deep learning methods for HAM10000 specifically could be more comprehensive to better position the paper's contribution. | Done. We would like to thank the reviewer for this insightful suggestion. We agree that the field of skin lesion classification is evolving rapidly, particularly with the emergence of Transformer-based and hybrid architectures.  In response to this comment, we have significantly updated Section 2 (Related Works) to reflect the most recent State-of-the-Art (SOTA) developments from 2024 and 2025. Specifically, we have added a new discussion highlighting the shift from pure CNNs to Vision Transformers (ViT) and Hybrid CNN-Transformer models. We have incorporated key recent studies, including:  Lian et al. (2025), regarding Shifted Windowing Vision Transformers.  Agarwal and Mahto (2025), regarding Hybrid CNN-Transformer models with Kolmogorov-Arnold Networks (CKAN).  Yang et al. (2025), regarding Multi-Scale Attention Boosting and Ensemble approaches.  This addition ensures that our proposed framework is positioned within the context of the latest architectural advancements in the field. |
| 14. The use of Avg-TopK pooling is presented as a key part of the framework. Since this is an existing method (Ref 10), its contribution to the final 1% accuracy gain (90.52% to 91.47%) should be analyzed more deeply. Is this small gain worth the added complexity? A comparison with standard Global Average Pooling should be included. | We thank the reviewer for this valuable comment. We would like to clarify that Global Average Pooling (GAP) and Avg-TopK belong to different pooling families: GAP is a global pooling operation applied at the last stage, whereas Avg-TopK is a local pooling operator applied inside intermediate convolutional blocks. In both of our configurations, the final GAP layer of Xception is preserved; in the Avg-TopK variant, only the intermediate max-pooling layers of the original Xception backbone are replaced by Avg-TopK pooling.  Therefore, the comparison in Table 6 is not a “GAP vs. Avg-TopK” replacement at the output, but rather “standard Xception with its default pooling stack + GAP” versus “Xception with local Avg-TopK pooling in place of intermediate max-pooling + GAP”. In this setting, the observed ~1% absolute improvement (from 90.52% to 91.47%) is consistent with the gains reported in the original Avg-TopK study and is practically relevant in an imbalanced medical classification task. |
| 15. The introduction should be updated to include more recent 2025 state-of-the-art literature concerning deep learning for skin cancer and other oncological applications to better emphasize the field's current successes and provide a more current context for the work.   - A novel hybrid ConvNeXt-based approach for enhanced skin lesion classification - A comprehensive comparison of convolutional neural network and visual transformer models on skin cancer classification - https://journals.adbascientific.com/aiapp/article/view/92 - https://journals.adbascientific.com/aiapp/article/view/89 - https://journals.adbascientific.com/aiapp/article/view/90 | Done.We sincerely thank the reviewer for this insightful comment. In accordance with this recommendation, the Introduction section has been carefully updated to include recent state-of-the-art studies published in 2025 that highlight current advances in deep learning for skin cancer and oncological image analysis. In particular, we have incorporated and discussed the following newly added references:  Aruk et al. (2025), Expert Systems with Applications, which proposes a novel hybrid ConvNeXt-based architecture for enhanced skin lesion classification, demonstrating the effectiveness of modern convolutional backbones in dermatological imaging.  Aruk et al. (2025), Computational Biology and Chemistry, presenting a comprehensive comparison between CNN and Vision Transformer models for skin cancer classification, offering an up-to-date benchmarking perspective.  Yang et al. (2025), Sensors, which introduces a multi-scale attention and ensemble Vision Transformer framework, emphasizing the role of attention mechanisms and model ensembling in improving classification robustness.  Lian et al. (2025), Clinics, which investigates a shifted-window Vision Transformer-based approach via transfer learning, highlighting the successful adaptation of transformer models to clinical skin cancer datasets.  These additions significantly strengthen the timeliness, relevance, and contextual depth of the Introduction by clearly demonstrating recent performance gains, architectural trends, and methodological directions in the field. Consequently, the revised manuscript now better reflects the current state-of-the-art (2025) and more clearly positions the proposed method within the contemporary research landscape. |

**Reviewer #2: The manuscript presents a promising lightweight approach to skin cancer classification integrating pruning, SMOTE, and Avg-TopK pooling. The topic is relevant, and the methodology is generally well-structured. The pruned Xception architecture achieves competitive performance and meaningful parameter reduction, which is valuable for deployment in constrained environments.**

| 1. Pruning methodology lacks robustness — sparsity is computed from a single test image, which may not represent general layer activation patterns. Consider evaluating sparsity across a validation batch. | Done. To address the reviewer’s concern regarding robustness, the sparsity computation has been updated. Instead of relying on a single test image, the sparsity values for all layers are now calculated using a validation batch of 16 images. This provides a more stable and representative activation profile and avoids biases introduced by image-specific activation patterns. The pruning decision (i.e., the selection of the layer with maximum sparsity) is now based on the averaged sparsity values across this validation batch.       |
| --- | --- |
| 2. Statistical validation is insufficient. Provide variance measures (CI, standard deviation) and statistical significance when comparing with benchmarks. | Done. Thank you for this valuable comment. In the revised manuscript, we addressed statistical validation in three ways:   - All performance tables were updated to report results as mean ± standard deviation over 5 independent runs (Tables 3–6).   This provides a variance-based assessment of model stability and allows fairer comparison with existing benchmarks. Although confidence intervals and hypothesis testing are uncommon in image-classification studies on HAM10000, the added variance measures already demonstrate that our improvements are consistent and not due to randomness. |
|        | |
| 3. Claims of clinical application are premature without real clinical trials or physician-validated evaluation. | Done. We agree with the reviewer that clinical applicability requires physician-validated evaluation and real clinical trials. In the revised manuscript, we removed any language that could be interpreted as a direct clinical claim and clarified that our model is intended solely as a research-stage computational framework. The Conclusion section was updated to explicitly state that the method is not yet clinically deployable and that future work will include collaboration with dermatologists and prospective clinical validation studies. The updated text in the revised manuscript is as follows:  “…Finally, although the proposed framework achieves strong quantitative performance, it is not intended to be interpreted as clinically deployable. Clinical applicability requires validation through dermatologist-supervised assessment, multi-center studies, and prospective clinical trials, which fall outside the scope of this study. Future research could extend this approach to other medical imaging tasks, broadening its applicability and impact in healthcare. …” |
| 4. Literature review is extensive but repetitive—some sections can be condensed. | Done. We thank the reviewer for this constructive suggestion. In response, the Related Works section has been carefully condensed to reduce redundancy while preserving all key technical content and references. Specifically, the descriptions of classical image processing methods (morphological operations, GLCM) and traditional machine learning approaches were streamlined to avoid repetition with Table~\ref{tab:related_works}. Additionally, ensemble learning discussions were refined to eliminate overlapping explanations. Importantly, the recent 2024–2025 deep learning and Vision Transformer-based studies were intentionally preserved in detail, as they are critical for contextualizing current state-of-the-art trends. These revisions improve clarity and readability while maintaining the comprehensive scope of the literature review. |
| 5. Numerous grammatical issues and awkward phrasing; manuscript needs language polishing. | Done. We thank the reviewer for highlighting the grammatical and phrasing issues. In response, the entire manuscript has been thoroughly revised for language quality, clarity, and readability. All sections—including the Abstract, Introduction, Methodology, Results, Discussion, and Conclusion—were carefully polished to correct grammatical errors, improve sentence structure, and ensure academic writing standards. These revisions are reflected in the updated manuscript. |
| 6. Figures should include clearer labels and higher resolution. | Done.We thank the reviewer for this helpful suggestion.  In response, all figures have been carefully revised to improve visual clarity and readability. Specifically, figure labels, axis titles, legends, and annotations were refined and standardized to ensure clear interpretation. In addition, all figures were regenerated and exported at a minimum resolution of 300 dpi, in accordance with journal publication standards. These improvements significantly enhance the overall quality and presentation of the figures in the revised manuscript. |
| 7. Ensure consistent notation across architecture sections. | Done. We carefully revised the entire Transfer Learning Models section to ensure a unified mathematical notation. Specifically, all architectures now use the same set of symbols:   - x: input feature map - h(l): output of layer l - W(l): learnable parameters of layer l - f(.): transformation function applied at a given layer   Furthermore, descriptions of depthwise–separable convolutions, residual connections, dense connections, and Inception multi-branch modules were rewritten using this unified notation system to ensure clarity and consistency throughout the manuscript. The revised version now reflects a coherent and standardized mathematical notation across all transfer learning model descriptions. |
| **Strengths**   1. Significant parameter reduction (35% fewer parameters) with minimal accuracy loss. 2. Well-structured experimental pipeline. 3. Novel application of Avg-TopK pooling improves feature retention. | We sincerely thank the reviewer for highlighting these strengths and for the positive evaluation of our methodological contributions, experimental design, and the effectiveness of the Avg-TopK pooling integration. |
